# Reduced Chemical Fertilizer Combined with Organic Fertilizer Alters the Soil Microbial Community and Enhances Soil Microbial Diversity of *Acanthopanax senticosus* Cultivation

**DOI:** 10.3390/microorganisms13122709

**Published:** 2025-11-27

**Authors:** Zhuolun Li, Xin Sui, Mengsha Li, Zhimin Yu, Pin Lv, Limin Wang, Jizhou Zhang, Wenqi Li

**Affiliations:** 1 Institute of Nature and Ecology, Heilongjiang Academy of Sciences, Harbin 150040, China; 19969675747@163.com (Z.L.); lvpin@haszrs.org.cn (P.L.); wanglimin0122@163.com (L.W.); jizhou1980229@126.com (J.Z.); liwenqi20000410@163.com (W.L.); 2 Engineering Research Center of Agricultural Microbiology Technology, Ministry of Education & Heilongjiang Provincial Key Laboratory of Ecological Restoration and Resource Utilization for Cold Region & Key Laboratory of Microbiology, College of Heilongjiang Province & School of Life Sciences, Heilongjiang University, Harbin 150080, China; xinsui_cool@126.com

**Keywords:** *Acanthopanax senticosus*, microorganisms, fertilization management, high-throughput sequencing

## Abstract

To investigate the response of soil microbial communities to reduce chemical fertilization supplementation with organic fertilizer in *Acanthopanax senticosus* cultivation, we analyzed the diversity, composition, and structure of soil microbiota by using high-throughput sequencing technology. The results showed that reducing chemical fertilizer application significantly increased soil microbial richness (ACE and Chao1 indices), which was positively correlated with soil total nitrogen (TN) content. At the phylum level, the relative abundance of Cyanobacteria decreased at T2 (reduction of 20% for fertilizer application) but increased at T4 (reduction of 60% for fertilization application), exhibiting an opposite trend to Bacteroidetes. At the genus level, the relative abundance of Paucibacter was significantly higher in T4 than in other treatments, while Nitrospira reached its peak under T3 treatment. For fungal communities, the richness index showed a non-linear response, initially decreasing and then increasing, which was positively correlated with the soil available potassium (AK) content. At the phylum level, reduced fertilizer application significantly reduced the relative abundance of Ascomycota compared to conventional fertilization. At the genus level, the relative abundance of Fusarium was significantly lower in the T4 treatment than in the other treatments. Redundancy analysis (RDA) revealed that the total organic carbon (TOC), TN, and AK were the key environmental factors affecting the soil microbial community. This study demonstrated that partial substitution of chemical fertilizers with organic amendments can improve soil physicochemical properties and enhance microbial diversity, providing a scientific basis for developing sustainable fertilization strategies for *Acanthopanax senticosus* cultivation.

## 1. Introduction

Heilongjiang Province is the largest commercial production region for *Acanthopanax senticosus* in China [1], with an annual cultivation area exceeding 13,000 ha, a dry product yield of ~28,000 tons (accounting for over 70% of the national market share), and an annual economic output of more than CNY 5 billion due to its high medicinal and health value derived from eleutherosides and polysaccharides [2]. Global chemical fertilizer use has reached 200 million tons annually [3]. While the application of chemical fertilizers enhances the *Acanthopanax senticosus* yield and bioactive compound content [4], long-term excessive use deteriorates soil physicochemical properties, resulting in agricultural non-point-source pollution and leading to environmental degradation [5,6]. Excessive nitrogen input, in particular, not only wastes resources but also leads to environmental pollution and soil degradation [7,8,9,10]. Consequently, sustainable cultivation practices are imperative to meet market demand and protect wild resources [11,12,13,14].

*Acanthopanax senticosus* has specific ecological requirements, thriving within a narrow temperature range (12–18 °C) and preferring loose, fertile, and well-drained soil [15, 16]. As a slow-growing plant, it takes 5–7 years to grow from planting to harvesting, necessitating sustained soil fertility. These constraints pose significant challenges to its cultivation, underscoring the need for scientifically sound soil management strategies.

Identifying reasonable fertilization practices that mitigate the environmental impacts of excessive fertilizer applications is therefore urgent. Integrated fertilization—combining reduced chemical fertilizer with organic fertilizer—has emerged as an environmentally friendly practice [17]. Appropriate organic substitution can enhance *Acanthopanax senticosus* yield, improve soil nutrient status, and protect soil ecology [18]. This approach reduces nutrient losses [19], increases fertilizer use efficiency, elevates soil organic matter [20], activates soil nutrients, changes soil enzyme activity, and enhances the content of bioactive compounds in *Acanthopanax senticosus* [21]. Furthermore, it enhances soil microbial activity, improves soil fertility, reduces soil salinization and soil-borne diseases [22], enhances root activity which refers to the physiological capacity of roots to absorb water/nutrients, synthesize hormones, and secrete exudates, reflected by root enzyme activities (e.g., nitrate reductase) and respiration rate [23], and boosts antioxidant enzyme activity and leaf pigment content [24]. It also increases water and fertilizer conservation and ultimately improves *Acanthopanax senticosus* yield and stress resistance.

Previous studies found that conventional fertilization combined with amino acid, fulvic acid, and biogas slurry yielded higher productivity and economic returns than conventional fertilization [25,26]. Bio-organic fertilizers outperformed conventional organic fertilizers in improving phosphorus use efficiency; replacing 40% of chemical fertilizer with bio-organic fertilizer significantly increased yield and active chemical ingredients increased yield by 12.3% and eleutheroside B content by 18.5%, while combining conventional fertilization with fulvic acid enhanced soil available nitrogen by 25.7% and reduced nutrient leaching by 19.8% [27]. Combined fertilization also increased the abundance of bacteria and actinomycetes, decreased the abundance of fungi, and increased activities of urease, catalase, sucrose, and alkaline phosphatase [28]. Organic substitution modifies microbiota via three mechanisms: (1) providing diverse carbon/nutrient sources favoring copiotrophs; (2) regulating soil pH and cation exchange capacity to optimize habitat; (3) introducing beneficial microbes that outcompete pathogens. Groups utilizing organic substrates increase, while those adapted to chemical fertilizers or unfavorable habitats decrease. A 30% reduction in nitrogen fertilization reduced soil electrical conductivity and improved nutrient content, leading to higher yield and bioactive compound content [29]. These findings confirm the economic and agronomic viability of reduced chemical fertilization supplemented with organic inputs.

However, few studies have focused on how such practices influence the soil microbial community structure and function in *Acanthopanax senticosus* systems. Microbial communities are sensitive indicators of soil health and are crucial in nutrient cycling and plant health [30, 31]. Soil microorganisms mediate core ecosystem functions such as nutrient cycling (nitrogen fixation, phosphorus solubilization), organic matter decomposition, and disease suppression, forming mutualistic interactions with plants: plants secrete root exudates to support microbial growth, while microorganisms improve nutrient availability and produce plant growth-promoting substances [32, 33]. For instance, in maize silage, increased nitrogen fertilization raised the abundance of aerobic bacteria, yeasts, and lactic acid bacteria but reduced mold abundance [34]. Similarly, in rice grains, appropriate nitrogen application promoted beneficial microbial colonization more effectively than either deficient or excessive application [35].

In this study, we conducted a pot experiment to investigate the effects of different fertilization regimes on soil nutrient dynamics and microbial community structure. We hypothesized that (1) reducing chemical fertilizer combined with organic fertilizer enhances soil nutrient availability; and (2) this practice significantly changes the structure and diversity of soil microbial communities.

## 2. Materials and Methods

### 2.1. Experimental Design and Soil Sampling

A pot experiment was conducted at the Institute of Nature and Ecology of Heilongjiang Academy of Sciences (126°65′ E, 45°71′ N). The study site has a temperate continental monsoon climate, with an average temperature of 15.6 °C, precipitation of 62.3 mm, and relative humidity of 68% in September 2024 (Heilongjiang Meteorological Bureau, 2024). Soil samples were collected at the harvest time of *Acanthopanax senticosus* in September 2024. Four fertilization treatments were established: T1, T2, T3, and T4. Each pot was preamended with a basal application of 40 g of fermented fungal chaff (rotted wood chips) and 120 g of decomposed cow manure, mixed with 6.6 kg of raw soil. T1 (conventional fertilization) received a total nutrient input equivalent to 1.5 kg of urea, 12 kg of ammonium phosphate, and 6 kg of potassium sulfate per 667 m^2^. T2, T3, T4 (reduced fertilization) received 20%, 40%, and 60% reductions in total chemical nutrients, respectively, based on the T1 regimen.

At harvest, soil samples were collected from each pot. After visible stones and plant residues were removed, soil was collected using a random sampling strategy: 5 subsamples (50 g each) from the rhizosphere (0–15 cm) of each pot were mixed into a composite sample (250 g per pot), then sieved through a 2 mm mesh. A total of 12 samples were analyzed, with 3 biological replicates per treatment (T1–T4). Each sample was divided into two subsamples: one was air-dried for the analysis of soil physicochemical properties, and the other was stored at −80 °C for subsequent DNA extraction and microbial community analysis.

### 2.2. Determination of Soil Physicochemical Properties

Soil physicochemical properties were determined according to the method described in [36]. Specifically: soil organic matter was determined by sulfuric acid oxidation with potassium chromate, soil alkaline nitrogen was determined by alkaline diffusion, soil available phosphorus was determined by the scandium molybdenum antimony cliometric method, and soil total nitrogen was determined by the Heyerdahl method.

### 2.3. DNA Extraction and PCR Amplification

According to the instructions, total soil DNA was extracted from 1 g of fresh soil using a soil DNA kit (Omega Bio-tek, Norcross, GA, USA). DNA was quantified by agarose gel electrophoresis (Bio-Rad, Hercules, CA, USA) and NANODROP (Thermo Fisher Scientific, Waltham, MA, USA), and all DNA concentrations were adjusted to 50 ng/μL and used for subsequent PCR. Bacterial PCR amplification was performed using universal primers 341F: 5′-ACTCCTACGGAGGCAGCA-3′; 806R: 5′-GGACTACHVGGGGTTTCTAAT-3′ for the V3–V4 region of the 16S ribosomal RNA gene [37], with TransStart FastPfu DNA polymerase. A 20 μL reaction system contained: 5× FastPfu Buffer (1× final concentration), dNTPs (0.25 mM each), forward/reverse primers (0.2 μM each), FastPfu DNA polymerase (1 U), template DNA (10 ng), ddH_2_O to 20 μL. The amplification procedure was: predenaturation at 95 °C for 2 min, followed by 25 cycles of amplification: 95 °C for 30 s, 55 °C for 30 s, 72 °C for 30 s, and finally extension at 72 °C for 5 min. The fungal PCR amplification was performed using primers ITS1F (F:CTTGGTCATTTAGAGGAAGTAA; R:GCTGCGTTCTTCATCGATGC) [38]. According to the instructions, PCR products were purified using the AxyPrep DNA Gel Extraction Kit (Axygen Biosciences, Union City, CA, USA). Purified PCR products were quantified using Qubit 3.0 (Life Invitrogen, Carlsbad, CA, USA) and amplicons from each of the 16 barcodes (GCACTTGCTAGATCGC). After adding the barcode to each PCR product, the mixed DNA products were used to prepare Illumina pair-end libraries according to the Illumina genomic DNA library preparation procedure. The amplicon libraries were sequenced on the Illumina NovaSeq 6000 platform (Illumina, San Diego, CA, USA) with 2 × 250 paired-end reads. Paired-end reads were assembled with a minimum overlap of 10 bp and maximum mismatch of 2 bp; final assembled read length was ~420 bp for bacteria and ~250 bp for fungi.

### 2.4. Bioinformatic Analyses

Raw sequencing data were processed using the QIIME2 pipeline (2023.5 distribution). Sequences were quality-filtered, denoised, and merged using the DADA2 plugin (reads with Phred quality score < 30, length < 200 bp, or ambiguous bases were removed; forward reads truncated at 240 bp, reverse reads at 200 bp using the DADA2 plugin) to infer amplicon sequence variants (ASVs), which provide a higher resolution than traditional OTU clustering [39]. Clusters of identical sequences let us detect microbial changes at fine-scale resolution. The phylogenetic affiliation of each 16S rRNA and ITS gene sequence was analyzed by uclust algorithm1 against the Silva (SSU138.1) 16S rRNA database using a confidence threshold of 80% [40]. Core microbiome was defined as ASVs present in ≥80% of samples within each treatment.

Subsequently, each sequence underwent taxonomic annotation using the RDP classifier and was aligned against the Silva 16S rRNA database (v138) with a similarity threshold of 80%. To acquire the taxonomic information corresponding to each ASV, the uclust algorithm was employed to conduct taxonomic analysis of the representative sequences of ASVs. Additionally, the community composition of each sample was quantified at all taxonomic levels, including domain, phylum, class, order, family, genus, and species. We have deposited our sequences in the SRA and the ID is 1362932.

### 2.5. Statistical Analysis

All statistical analyses were performed using the BMKCloud online platform (https://www.biocloud.net/ accessed on 15 October 2024) and SPSS software (version 26.0, IBM Corp., Armonk, NY, USA). One-way analysis of variance (ANOVA) followed by Tukey’s honest significant difference (HSD) post hoc test was used to determine significant differences (*p* < 0.05) in soil properties and alpha-diversity indices among treatments. Alpha-diversity within samples was assessed using the Chao1 and ACE (richness) and Shannon and Simpson (diversity) indices by using QIIME2 2020.6 software [41]. Rarefaction curves were generated to evaluate sequencing depth. Beta-diversity between samples was analyzed based on Bray–Curtis dissimilarity matrices and visualized via principal coordinates analysis (PCoA). Permutational multivariate analysis of variance (PERMANOVA) was used to test for significant differences in microbial community structure among groups [42]. Differential abundance analysis of taxa (biomarkers) between specific groups was identified using statistical methods such as linear discriminant analysis effect size (LEfSe) or the Kruskal–Wallis test [43]. The Kruskal–Wallis test was used for non-normally distributed taxonomic abundance data; LEfSe identified biomarkers with LDA > 2.0 and *p* < 0.05.

## 3. Results

### 3.1. Changes in Soil Physicochemical Properties Under Different Fertilization Treatments

All soil physicochemical properties except available potassium (AK) showed significant differences among treatments (*p* < 0.05) (Table 1). Soil pH exhibited a gradient decrease as fertilizer application was reduced, showing significant differences across all treatments (*p* < 0.05). The T4 treatment (60% reduction) showed the most pronounced effects on enhancing soil nitrogen and organic carbon accumulation. Its alkaline dissolved nitrogen (AN: 294.47 ± 21.40 mg/kg) and organic carbon (TOC: 2.967 ± 0.002 g/kg) were significantly higher than those in all other treatments (*p* < 0.05). Total nitrogen content (TN: 0.214 ± 0.001 g/kg) in T4 also reached the highest value. In contrast, the conventional fertilization treatment (T1) resulted in the highest content of available phosphorus (AP: 53.29 ± 2.56 mg/kg) and available potassium (AK: 222.51 ± 17.35 mg). Notably, the T2 treatment (20% reduction) appeared to suppress total nutrient levels, showing the lowest values for TN (0.183 ± 0.001 g/kg), TOC (2.685 ± 0.002 g/kg), and AP (38.47 ± 0.24 mg/kg).

### 3.2. Soil Microbial α- and β-Diversities Under Different Fertilization Treatments

The dilution curves reflect the rate of new species emergence under continuous sampling, with the rate of new species discovery decreasing rapidly in all sample groups as the sequencing depth increases and gradually converging to a plateau period after reaching a certain sequencing volume. In addition, T3 exhibited the highest number of OTUs (3390), whereas only 2193 OTUs were identified in T2, suggesting that the sequencing depth used in this study adequately covered the microbial diversity within each sample group.

**Figure 1:**
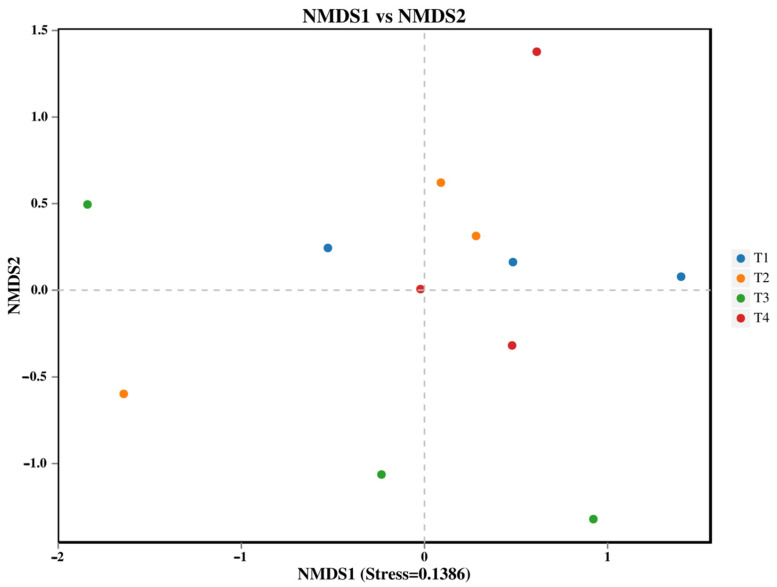
NMDS analysis of soil bacteria: each point in the graph represents a sample; different colors represent different groups.

The α-diversity indices (including Chao1, ACE, Shannon, and Simpson) of both bacterial and fungal communities showed no significant differences among the various fertilization treatments (Table 2). In contrast, the β-diversity of the soil bacterial community, analyzed using non-metric multidimensional scaling (NMDS), was significantly altered by the fertilization regimes (Figure 1, PERMANOVA, *p* < 0.05). Specifically, the bacterial community structure in the T3 treatment (40% reduction) was significantly distinct from those in both the T1 (conventional fertilization) and T4 (60% reduction) treatments.

**Figure 2:**
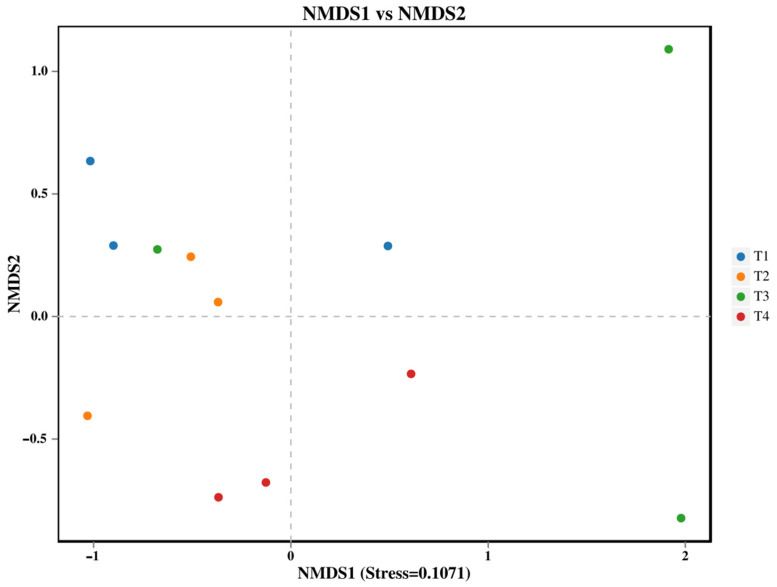
NMDS analysis of soil fungi: each point in the graph represents a sample; different colors represent different groups.

Soil fungal β-diversity changed significantly in different fertilization applications (Figure 2, PERMANOVA *p* < 0.05). T1 treatment differed significantly compared to T4 treatment.

### 3.3. Soil Microbial Composition in Response to Different Fertilization Measures

**Figure 3:**
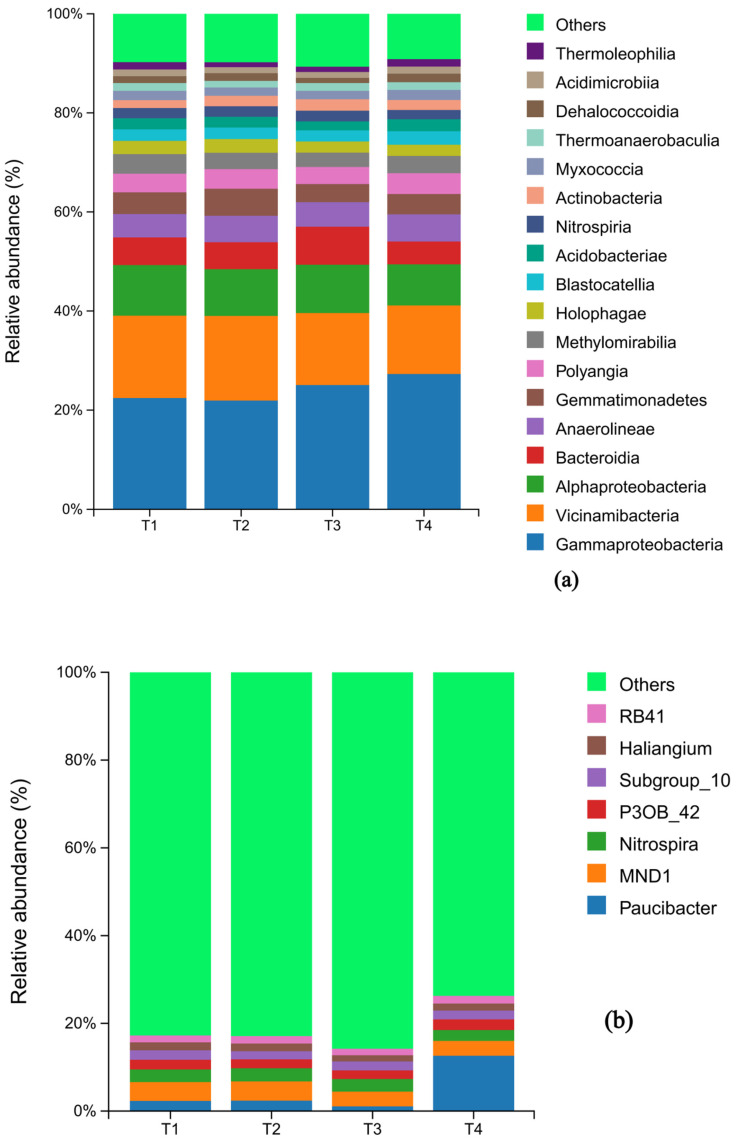
Histogram of the distribution of bacteria species under different fertilization levels: the horizontal axis is the name of the sample; the vertical axis is the percentage of relative abundance; different colors indicate different species. (**a**) shows the composition of soil bacteria at the phylum level with abundance greater than 1%, while (**b**) shows the composition of soil bacteria whose abundance ranked in the top 20 at the genus level.

The composition of both bacterial and fungal communities was significantly influenced by the fertilization treatments. At the phylum level, the bacterial communities across all treatments were predominantly composed of Proteobacteria (29.0%, 28.1%, 31.3%, 31.7%), Acidobacteria (22.9%, 23.4%, 20.5%, 20.7%). Chloroflexi and Proteobacteria also represented substantial proportions of the community. A notable shift was observed in response to fertilizer reduction: the relative abundance of Bacteroidota decreased initially and then increased, reaching its minimum at T2. Conversely, the abundance of Bacteroidota increased and then decreased, peaking at T2. In contrast, the relative abundances of Myxococota and Actionbacteriota remained relatively stable across treatments (Figure 3a).

At the genus level of bacteria, the relative abundance of Bacteroidea changed extremely significantly (1.47%, 1.49%, 0.73%, 8.41%) with the reduction of fertilizer additions, showing a decreasing and then significantly increasing trend, which was significantly higher in the T4 group than in the other treatments; the abundance of the genera of Chloroform and Gemmatimonadaceae first increased and then decreased and was significantly higher in the T2 group than other treatments (*p* < 0.05); Vicinamibacterales abundance showed a decreasing trend; while Nitrospira abundance had the maximum peak (1.94%) under T3 treatment (Figure 3b).

At the genus level, the relative abundance of Paucibacter changed most dramatically, exhibiting a pattern of decrease followed by a significant increase (T1: 1.47%, T2: 1.49%, T3: 0.73%, T4: 8.41%). Its abundance in the T4 treatment was significantly higher than in all other groups (*p* < 0.05). The abundance of Gemmatimonas and bacteria from the family Gemmatimonadaceae first increased and then decreased, with the T2 treatment showing a significantly higher abundance than others. The abundance of Vicinamibacteraceae showed a general decreasing trend across the treatment gradient. Notably, the abundance of the nitrifying genus Nitrospira reached its maximum (1.94%) under the T3 treatment (Figure 3b).

**Figure 4:**
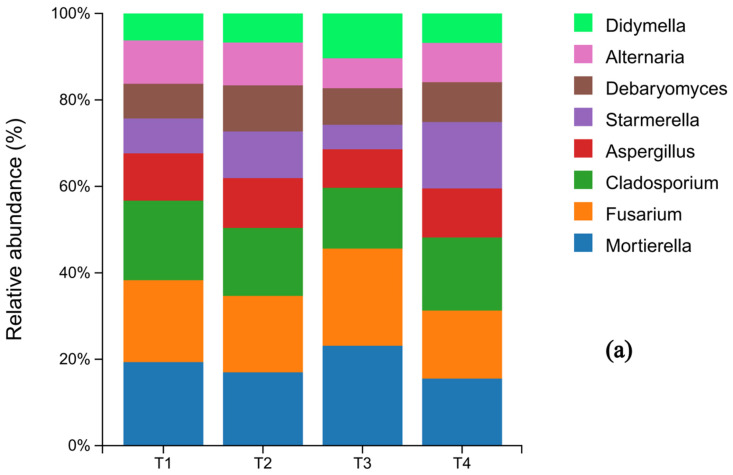

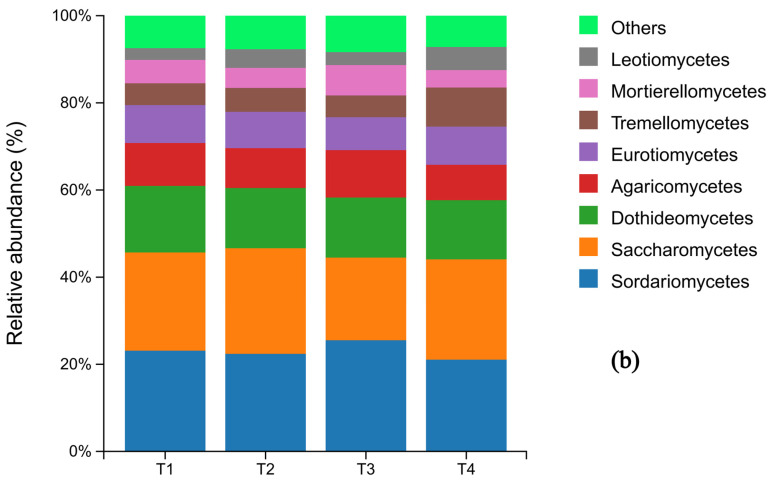
Histograms of fungal species distribution under different fertilization levels (N = 3 pretreatment): sample names on the horizontal axis; percentage of relative abundance on the vertical axis; different colors indicate different species. (**a**) shows the composition of soil fungi at the phylum level for the top 10 species in terms of abundance, while (**b**) shows the composition of soil fungi whose abundance ranked among the top 10 at the genus level.

At the phylum level of fungi, Ascomycota was the dominant phylum across all treatments, representing 70.4%, 70.6%, 67.1%, and 69.9% of the communities in T1 through T4, respectively. Notably, its relative abundance in the T3 treatment was lower than in the other treatments. Basidiomycota was the second most abundant phylum. The relative abundances of other fungal phyla remained largely unchanged across the fertilization gradient (Figure 4a). Significant shifts were observed at the genus level. The relative abundance of Mortierella varied considerably (T1: 4.61%, T2: 3.89%, T3: 5.39%, T4: 3.42%), with its abundance in the T4 treatment being significantly lower than in all other treatments. In contrast, the abundance of Cladosporium was highest under the conventional fertilization treatment (T1) compared to the reduced fertilization groups (Figure 4b).

### 3.4. Relationships Between Soil Microbial Communities and Soil Physicochemical Properties

**Figure 5:**
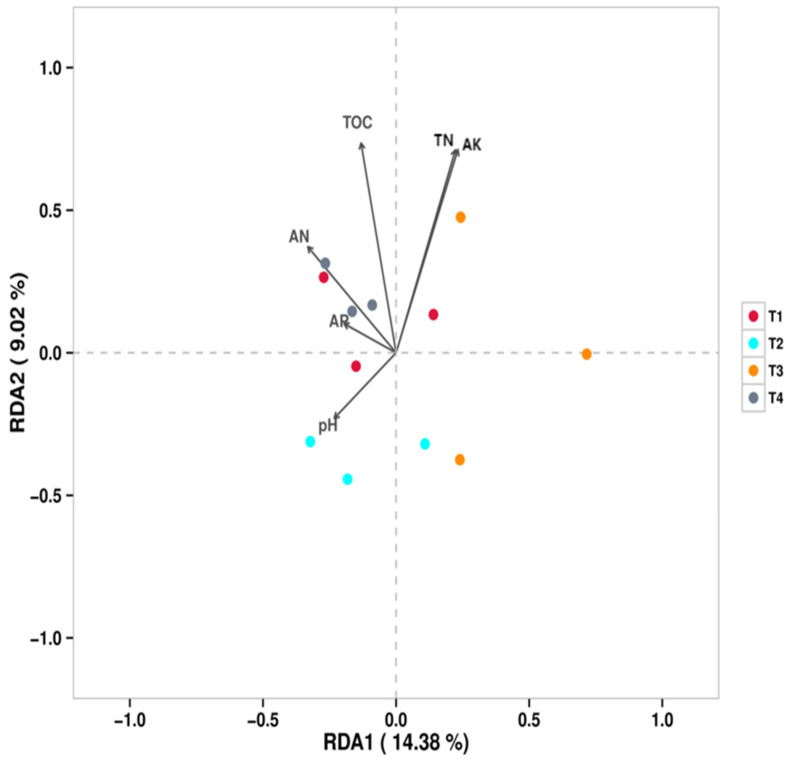
RDA/CCA—Sample Points (bacteria).

Redundancy analysis (RDA) was performed to elucidate the relationships between soil bacterial communities (genus level) and environmental variables (Figure 5). The results indicated that total organic carbon (TOC) was the strongest environmental factor explaining the variations in bacterial community structure under different fertilization regimes. The distribution of bacterial communities from different treatments along the RDA axes revealed distinct correlations with soil properties: the bacterial community in the T1 (conventional fertilization) treatment was positively correlated with total nitrogen (TN), available nitrogen (AN), and available potassium (AK) but negatively correlated with pH. The bacterial community in the T2 (20% reduction) treatment showed a positive correlation with pH but negative correlations with AK and TOC. The bacterial community in the T4 (60% reduction) treatment was positively correlated with AN, TN, and TOC.

**Figure 6:**
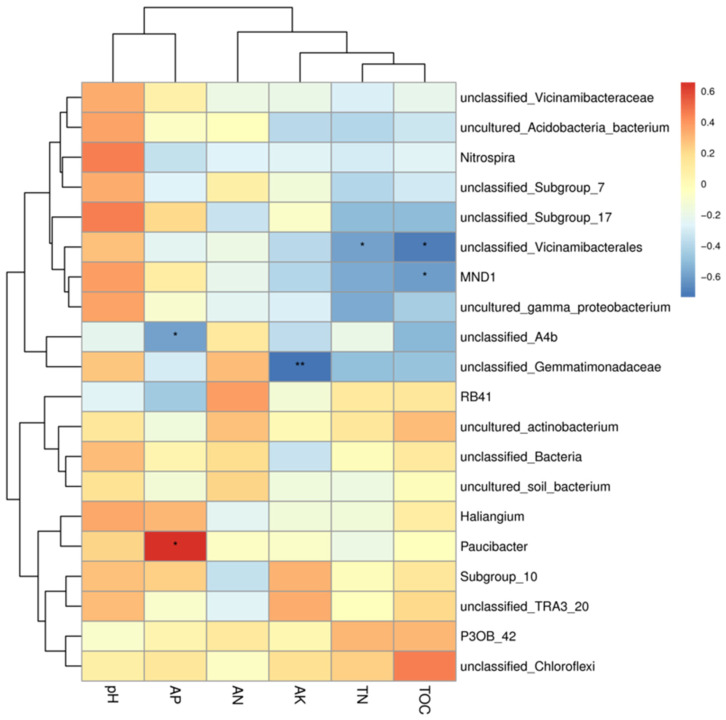
Correlation diagram between soil physical and chemical properties and microbial species. The left clustering tree is the species clustering tree, the upper clustering tree is the sample clustering tree, and the middle is the heat map. If sample clustering information is available, the color corresponds to the legend, red is positive correlation, blue is negative correlation, and a darker color indicates higher correlation; * indicates significant correlation (* is <0.05, ** is <0.001).

A heat map of the correlation between the 20 most abundant bacterial genera and soil physicochemical properties was made (Figure 6), in which the main genera in the bacterial community were Nitrospira, Paucibacter, and MND1. While Paucibacter showed a strong correlation with AP, Chloroflexi showed a stronger correlation with TOC, and Nitrospira, MND1, and pH also showed a positive correlation. MND1 was also correlated with pH.

A heat map was constructed to visualize the Spearman correlation coefficients between the relative abundance of the top 20 most abundant bacterial genera and the soil physicochemical properties (Figure 6). Nitrospira exhibited a significant positive correlation with soil pH. Paucibacter showed a strong positive correlation with available phosphorus (AP). Chloroflexi (representing a phylum; the dominant genus within it should be specified if possible) was strongly positively correlated with total organic carbon (TOC). MND1 (a candidate genus) was positively correlated with pH.

**Figure 7:**
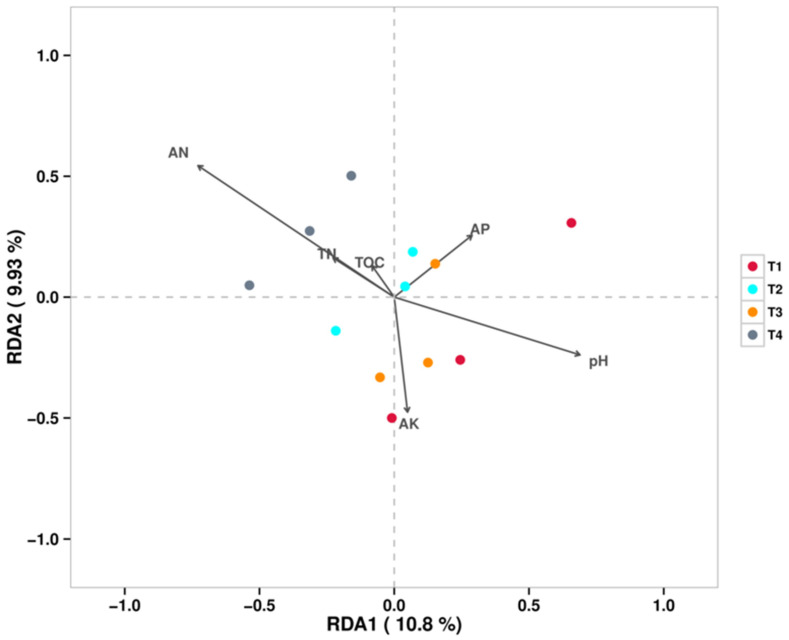
RDA/CCA (Fungi).

Figure 7 shows the RDA/CCA at the fungal genus level, which indicated that the strongest factor contributing to the differences in soil fungal communities under different fertilization conditions was AN. The soil fungal communities of all T4 treatments were positively correlated with AN and negatively correlated with pH, while Fusarium abundance was positively correlated with pH.

### 3.5. Significance Analysis of Differences Between Soil Microbiomes of Different Fertilization Practices

**Figure 8:**
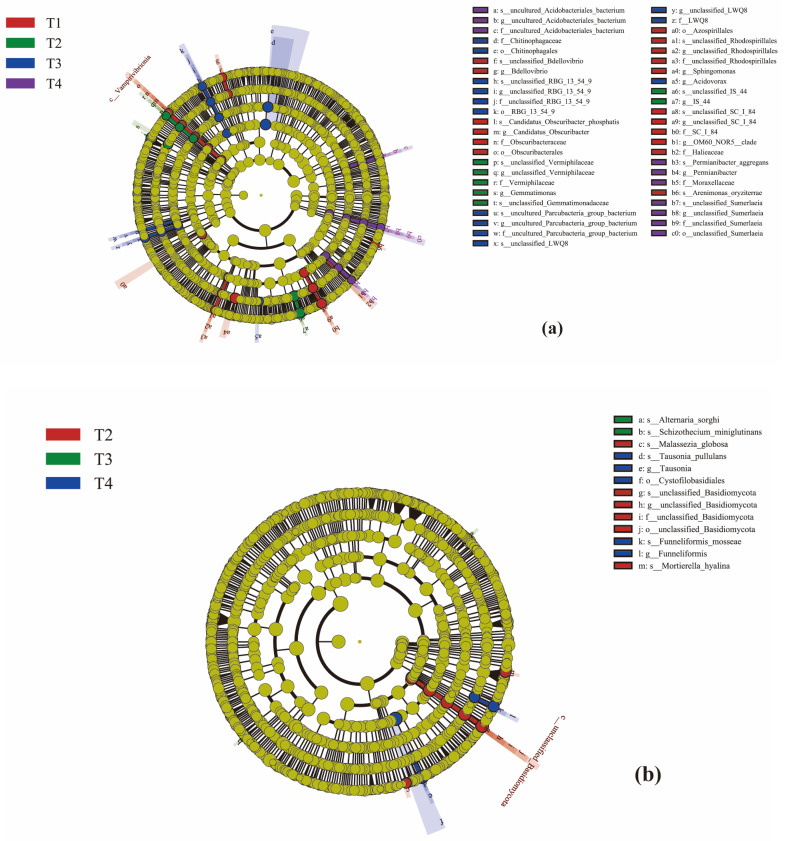
Evolutionary branch diagram. The circles radiating from the inside to the outside represent the taxonomic level from phylum to species; Each small circle at a different taxonomic level represents a classification at that level, and the diameter of the small circles is proportional to the relative abundance; The coloration principle is that the species with no significant differences are uniformly colored yellow, and the other species are colored according to the group with the highest abundance of the group. Different colors represent different groups, and nodes of different colors represent microbiota that play an important role in the grouping represented by that color. (**a**) shows bacteria and (**b**) shows fungi.

Line discriminant analysis (LDA) effect size (LEfSe) analysis was able to search for statistically different biomarkers between groups (Figure 8a,b). Under the four treatments, Figure 8a shows differences in 47 bacterial taxa, where LDA > 2.0, and Figure 8b shows differences in 13 fungal taxa, but only in T2, T3, and T4, where LDA > 2.0.

RDA showed that TOC, TN, and AN explained 15.6%, 12.3%, and 10.1% of bacterial community variation, respectively (total explained variance = 38.0%). For fungi, AN and pH explained 10.8% and 9.5% of community variation (total explained variance = 20.3%). LEfSe analysis identified 47 bacterial biomarkers (maximum LDA score = 4.2) and 13 fungal biomarkers (maximum LDA score = 3.8) across treatments.

## 4. Discussion

### 4.1. Dual Effects of Fertilizer Application on Soil Fertility

Long-term over-application of chemical fertilizers leads to soil structure degradation, nutrient imbalance, and declines in enzyme activity, which can inhibit crop physiological metabolism and growth, thereby threatening the sustainable use of soil resources [44,45,46, 47]. In this study, although the high fertilizer application rate (T1) increased the contents of available phosphorus (AP) and available potassium (AK) (53.29 mg/kg and 222.51 mg/kg, respectively) in the short term, it also induced a significant increase in pH (7.74). This alkalization may inhibit the microbial activity in naturally acidic soils, consistent with previous studies showing that excessive fertilizer leads to soil acidification or salinization [48].

In contrast, the low fertilization rate (T4) optimized long-term soil fertility sustainability. It promoted organic matter decomposition (resulting in a TOC of 2.967 g/kg) and achieved the highest total nitrogen (TN) content (0.214 g/kg) among all treatments. This indicates that reducing chemical fertilizer input while incorporating organic matter can effectively enhance soil carbon and nitrogen sequestration capacity, providing a more stable energy source and substrates for microbial activities [49]. These results verify the advantages of organic–inorganic fertilization in maintaining soil fertility sustainability [50], which is particularly crucial for slow-growing perennial plants like Acanthopanax that require long-term stable nutrient supply.

Furthermore, the available nitrogen (AN) content under the T4 treatment (294.47 mg/kg) was significantly higher than in other treatments (ranging from 139.23 to 155.27 mg/kg). This suggests that reducing chemical fertilizer combined with organic substrates (e.g., fungal chaff, decomposed cow manure) may more efficiently promote soil organic nitrogen mineralization and/or biological nitrogen fixation. On one hand, lower chemical nitrogen input may reduce the nitrification inhibition effect, potentially enhancing the activity of ammonia-oxidizing bacteria (e.g., Nitrospira) (Figure 4b). On the other hand, organic matter input likely stimulated the functional expression of nitrogen-fixing and organic-nitrogen-mineralizing bacteria [51]. This aligns with previous observations in rice studies, where appropriate nitrogen fertilization enhanced microbial colonization to improve seed quality.

### 4.2. Effect of Fertilization Rate on Microbial Diversity and Structure

Soil microorganisms are one of the key factors in soil nutrient transformation, and their diversity and community structure represent the metabolic capacity of soil ecosystems [52]. In our analysis of bacterial and fungal diversity, the reduction in fertilizer application did not lead to obvious differences in diversity indices, which contrasts with the findings of Nie et al. [53]. This discrepancy may be related to the relatively short duration of our experiment (one year).

Notably, all four diversity indices for the fungal community were highest in the T3 treatment. In contrast, the Shannon index of the bacterial community showed a positive correlation with fertilizer application rate, indicating enhanced community evenness under high-fertilizer conditions. This may stem from excess fertilizer stimulating the explosive growth of a few eutrophic bacterial taxa (e.g., Proteobacteria) to form a dominant community (Figure 3a). Similarly, studies on silage maize have found that increased nitrogen fertilizer application alters microbial community structure. However, the Chao1 and ACE indices, reflecting species richness, were lowest in the T2 treatment (20% reduction) and highest in T4 (60% reduction). This suggests that medium-level fertilization (T2) may create ecological stress, where invading populations compete with native species for resources, forcing some microbial taxa to narrow their niche breadth. Conversely, low-level fertilization (T4) combined with organic inputs appeared to create a more heterogeneous environment that facilitated the recovery and coexistence of rare species.

In the microbial community structure analysis, the bacterial phyla Proteobacteria and Acidobacteria emerged as dominant groups, collectively accounting for over 50% of the community and serving as the core drivers of soil nutrient cycling [54,55]. Their abundance variations reflect changes in soil nutrient cycling capacity. As fertilizer application decreased, the abundance of Proteobacteria (typically copiotrophic) showed a “V”-shaped trend (lowest in T2), while Acidobacteria (typically oligotrophic) exhibited an inverted “V”-shaped trend (highest in T2). The T2 treatment (20% reduction) may have created a “transition pressure,” inhibiting copiotrophic Proteobacteria while transiently promoting oligotrophic Acidobacteria. The 60% reduction in T4, coupled with organic matter supplementation, may have helped re-establish a balance between these two phyla.

The enrichment of Sphingomonas and Gemmatimonas in the T4 group is noteworthy, as these genera are known to promote the synthesis of bioactive compounds like sphingans and can directly enhance medicinal plant quality, aligning with previous studies [56]. In contrast, the over-expansion of Acidobacteria in T2 may be related to decreased soil carbon metabolism efficiency. Additionally, the increase in methanol-oxidizing bacteria in the T3 group suggests that this fertilization rate might optimize carbon source utilization pathways and reduce energy loss.

The genus Paucibacter showed an increasing trend in relative abundance with fertilizer reduction, reaching its highest level in the T4 treatment (Figure 3b). Paucibacter has been reported to participate in organic matter degradation (e.g., aromatic compounds) and possesses plant-beneficial potential [57,58]. Its dominance in low-fertilizer, high-organic-matter environments (T3, T4) suggests a key role in carbon cycling and plant-root interactions.

The fungal community was primarily dominated by the phyla Ascomycota and Basidiomycota, consistent with previous reports [59, 60], with Trichoderma, Fusarium, and Cladosporium as the top three dominant genera. Ascomycota are a major component of the root-associated soil mycobiome, mostly comprising saprophytes that decompose organic matter and play vital roles in nutrient cycling. However, this phylum also contains many plant pathogens like Fusarium, which causes significant damage through diseases like tobacco wilt and root rot [61,62]. Basidiomycota are particularly efficient in decomposing lignocellulose, converting plant residues into plant-available nutrients [63]. In this study, the abundance of Fusarium was significantly lower in T3 and T4 treatments compared to other groups. This reduction may be related to Fusarium’s preference for low-nutrient soils, as its abundance has been shown to be negatively correlated with soil nitrogen content [64]. Specifically, the significant reduction of Fusarium in T4 aligns with findings that a 20% reduction in conventional nitrogen application with microbial inoculants could effectively reduce Fusarium abundance in tobacco soils [65]. Similarly, Ding Jianing [66] found that organic fertilizer substitution could reduce the relative abundance of Ascomycota. These results collectively suggest that reduced fertilizer application promotes soil nutrient cycling and reduces the risk of pathogen proliferation.

Notably, diverse soil microbial communities shaped by organic substitution can not only regulate nutrient cycling but also degrade harsh pesticides (e.g., herbicides, insecticides) through enzymatic decomposition, enhancing soil detoxification capacity and resilience [67, 68]. This further supports the ecological value of the optimized fertilization regime for long-term *A. senticosus* cultivation.

Although high-throughput amplicon sequencing (metabarcoding) effectively characterized taxonomic shifts in the *Acanthopanax senticosus* soil microbiome under different fertilization regimes, this approach has recognized limitations. PCR amplification and sequencing biases, incomplete reference databases, and the inclusion of extracellular or relic DNA may influence diversity estimates and taxonomic resolution. Critically, metabarcoding provides no direct insight into microbial functionality or metabolic activity. Future studies integrating shotgun metagenomics, metatranscriptomics, or soil metabolomics are recommended to elucidate functional responses and link community structure to ecosystem processes.

### 4.3. Effects of Soil Properties on Microbial Communities

Both correlation heat map and redundancy analysis (RDA) of the bacterial community clearly indicated that total organic carbon (TOC) was the strongest environmental factor driving bacterial community differences. The T4 treatment showed strong positive correlations with TOC, AN, and TN. This strongly suggests that the low fertilizer input combined with high-organic-matter amendments drove microbial community assembly toward a composition potentially more functionally adapted to organic matter degradation and nutrient cycling, shaped by the unique soil nutrient environment created by this management practice. This is consistent with findings from Wang et al. [69] in mango cultivation and Sun et al. [70] in tobacco fields, where reduced chemical fertilizer combined with organic amendments improved both soil microbial communities and physicochemical properties. LEfSe analysis (Figure 8) further identified that the T4-treated plots contained the highest number of significantly differentiated bacterial and fungal biomarkers, including enrichments of the Chloroflexi phylum and Paucibacter genus. This statistically confirms the uniqueness of the T4 microbial community and demonstrates the progressive shaping of microbial communities along the fertilization gradient. In the RDA of the fungal community, available nitrogen (AN) emerged as the strongest factor influencing fungal community structure. The pathogenic genus Fusarium showed a positive correlation with pH, while its abundance significantly decreased in the T4 group with reduced fertilizer application. This further illustrates that reduced fertilizer application decreases the abundance of pathogenic genera, contributing to a healthier soil ecosystem.

In addition, the identified microbial biomarkers fulfill critical ecological and functional roles in sustaining soil health for *Acanthopanax senticosus* cultivation: Paucibacter (enriched in T4) drives complex organic matter decomposition, enhancing carbon sequestration and nutrient supply. Nitrospira (peaking in T3) mediates nitrification, optimizing nitrogen availability and reducing losses. Reduced Fusarium abundance in T3/T4 mitigates soil-borne disease risk, while Basidiomycota supports recalcitrant residue decomposition and habitat heterogeneity. These biomarkers link fertilization regimes to key functions—nutrient cycling, organic matter turnover, and disease suppression—underpinning sustainable cultivation.

In summary, in T1 (conventional fertilization), the abundant inorganic fertilizers make microorganisms preferring fast-acting nutrients (such as Proteobacteria) become dominant groups; the relatively high pH inhibits acidophilic microorganisms and provides a suitable growth condition for the pathogenic Fusarium. In T2 (20% fertilizer reduction), the insufficient fast-acting nutrients lead to an increase in acidophilic microorganisms adapted to low-nutrient environments (such as Acidobacteria) [71], a decrease in microorganisms requiring complex nutrients, and the loss of rare microorganisms due to single nutrient conditions, with little change in the fungal community [72, 73]. For T3 (40% fertilizer reduction), the balanced nutrient supply makes nitrifying bacteria capable of decomposing ammonia (such as Nitrospira) reach the highest quantity; among fungi, groups that decompose lignin (such as Basidiomycota) increase while pathogenic Fusarium decreases, and bacteria and fungi can also cooperate to utilize nutrients more efficiently. As for T4 (60% fertilizer reduction), the rich organic matter makes microorganisms good at decomposing complex organic matter (such as Paucibacter) account for the highest proportion; the diverse microenvironments allow rare microorganisms to reappear, and at the same time, the high nutrient content and relatively low pH inhibit the growth of pathogenic Fusarium [74, 75].

## 5. Conclusions

This study elucidates the mechanisms through which fertilizer application rates have an influence. Through comprehensive analysis of soil fertility, microbial diversity, and their correlations, we demonstrate that reduced chemical fertilizer application combined with organic amendments represents an optimal strategy for balancing ecological sustainability and production efficiency in *A. senticosus* cultivation. This approach reshaped soil microbial diversity, community structure, and functional potential, thereby optimizing nutrient cycling efficiency within the rhizosphere microenvironment. This microbiome-centric soil management strategy not only alleviated the stress of over-fertilization on microbial community stability but also provides novel insights for enhancing medicinal plant quality through rhizosphere engineering. Future research should focus on long-term field experiments to validate the dynamic effects of this fertilization regime on the accumulation of bioactive compounds in *Acanthopanax senticosus* roots. Such studies would provide a scientific foundation for precision soil management in medicinal plant cultivation.

## Figures and Tables

**Table 1:** Soil chemical properties under each treatment. AP, available phosphorus; AK, available potassium; AN, hydrolytic nitrogen; TN, total nitrogen; TOC, total organic carbon.

	pH	AP (mg/kg)	AK (mg/kg)	AN (mg/kg)	TN (g/kg)	TOC (g/kg)
T1	7.74 ± 0.02 a	53.29 ± 2.56 a	222.51 ± 17.35 a	139.23 ± 3.59 b	0.201 ± 0.001 c	2.937 ± 0.002 b
T2	7.58 ± 0.006 b	38.47 ± 0.24 b	180.18 ± 2.65 a	155.27 ± 3.25 b	0.183 ± 0.001 d	2.685 ± 0.002 d
T3	7.44 ± 0.03 c	45.58 ± 6.82 ab	214.57 ± 2.65 a	150.78 ± 3.96 b	0.207 ± 0.001 b	2.823 ± 0.002 c
T4	7.41 ± 0.003 c	49.10 ± 0.73 ab	196.05 ± 14.01 a	294.47 ± 21.40 a	0.214 ± 0.001 a	2.967 ± 0.002 a

Note: Significant differences (*p* < 0.05) are indicated by the different letters in each column.

**Table 2:** Soil microbial α-diversity index under different fertilization measures.

	Diversity Index	CK	T2	T3	T4
Bacteria	Shannon	10.43 ± 0.09 a	10.39 ± 0.16 a	10.40 ± 0.31 a	9.98 ± 0.99 a
	Simpson	0.99 ± 0.002 a	0.99 ± 0.001 a	0.99 ± 0.001 a	0.98 ± 0.03 a
	ACE	2646.86 ± 15.54 a	2535.10 ± 396.93 a	2691.27 ± 624.02 a	2901.77 ± 336.57 a
	Chao1	2644.74 ± 15.58 a	2532.28 ± 396.53 a	2687.69 ± 623.65 a	2897.67 ± 336.85 a
	ASVs	2531.67 ± 9.02 a	2644.33 ± 228.82 a	2687 ± 360.16 a	2897 ± 194.54 a
Fungal	Shannon	7.91 ± 0.17 a	7.82 ± 0.12 a	7.99 ± 0.34 a	7.77 ± 0.27 a
	Simpson	0.97 ± 0.006 a	0.97 ± 0.002 a	0.98 ± 0.009 a	0.97 ± 0.005 a
	ACE	601.03 ± 67.97 a	587.63 ± 34.26 a	608.24 ± 96.83 a	585.79 ± 54.75 a
	Chao1	600.69 ± 68.21 a	587.33 ± 34.27 a	608.05 ± 96.99 a	585.67 ± 54.85 a
	ASVs	600.67 ± 68.22	587.33 ± 34.27	608 ± 97.04	585.67 ± 54.85

Note: Significant differences (*p* < 0.05) are indicated by the different letters in each column.

## Data Availability

The original contributions presented in this study are included in the article/Supplementary Material. Further inquiries can be directed to the corresponding authors.

## Supplementary Materials

The following supporting information can be downloaded at: https://www.mdpi.com/article/10.3390/microorganisms13122709/s1. Table S1. Fertilization Treatments. Figure S1. Rarefaction Curves. Figure S2. UPGMA clustering analysis (Bacteria). Figure S3. UPGMA clustering analysis (Fungal). Table S2. Microbial biomarkers for each fertilization treatment with columns for treatment, bacterial biomarkers, and fungal biomarkers.
