# Supplementary material for: Reduced Chemical Fertilizer Combined with Organic Fertilizer Alters the Soil Microbial Community and Enhances Soil Microbial Diversity of Acanthopanax senticosus Cultivation
Source: Microorganisms. 2025 Nov 27;13(12):2709. doi: 10.3390/microorganisms13122709 (PMC12735039; doi:10.3390/microorganisms13122709)
Supplement: Supplementary file 1 [file microorganisms-13-02709-s001.zip › microorganisms-3918445-supplementary.pdf]

Table S1. Fertilization Treatments

Treatment	Fertilizer Regime	Chemical Fertilizer Reduction Rate	Replicates
T1	Conventional fertilization (1.5 kg urea + 12 kg ammonium phosphate + 6 kg potassium sulfate per 667 m^2)	0%	3
T2	Reduced fertilization based on T1	20%	3
T3	Reduced fertilization based on T1	40%	3
T4	Reduced fertilization based on T1	60%	3

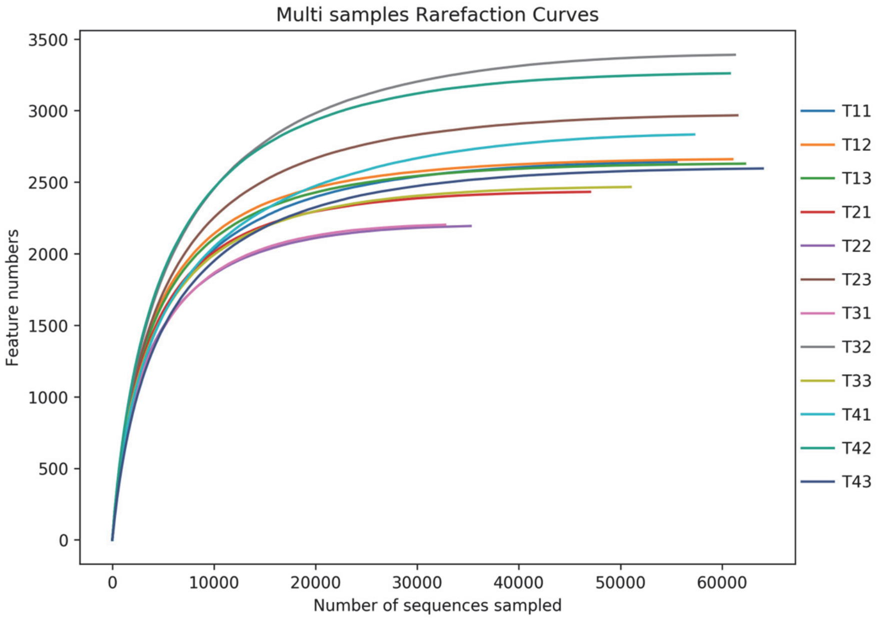

Figure S1. Rarefaction Curves.

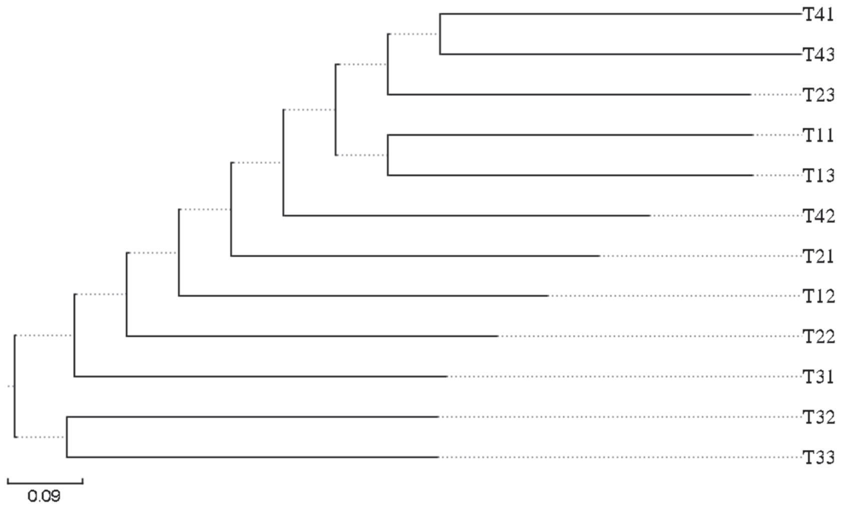

Figure S2. UPGMA clustering analysis(Bacteria).

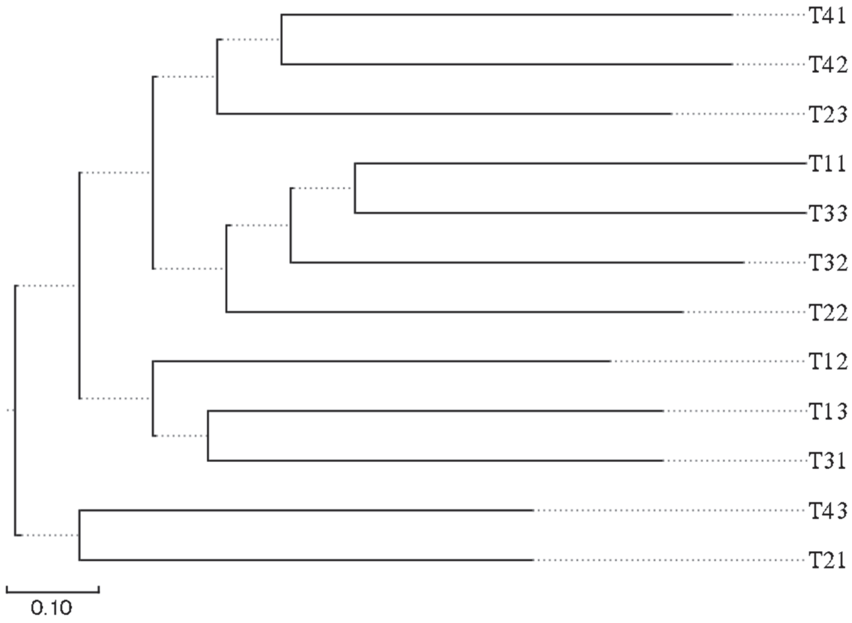

Figure S3. UPGMA clustering analysis(Fungal).

Table S2. Microbial biomarkers for each fertilization treatment with columns for treatment, bacterial biomarkers, and fungal biomarkers.

Treatment	Bacterial Biomarkers	Fungal Biomarkers
T1	Gemmatimonadaceae (genus), Vicinamibacterales (order)	Cladosporium (genus), Aspergillus (genus)
T2	Acidobacteria (phylum), Subgroup_7 (genus)	Mortierella (genus), Mucoromycota (phylum)
T3	Nitrospira (genus), Nitrospirota (phylum)	Basidiomycota (phylum), Debaryomyces (genus)
T4	Paucibacter (genus), Chloroflexi (phylum)	None significant
